# Atomic structure and electronic properties of MgO grain boundaries in tunnelling magnetoresistive devices

**DOI:** 10.1038/srep45594

**Published:** 2017-04-04

**Authors:** Jonathan J. Bean, Mitsuhiro Saito, Shunsuke Fukami, Hideo Sato, Shoji Ikeda, Hideo Ohno, Yuichi Ikuhara, Keith P. McKenna

**Affiliations:** 1 Department of Physics, University of York, Heslington, York, North Yorkshire, YO10 5DD,UK; 2 Institute of Engineering Innovation, The University of Tokyo, 2-11-16 Yayoi, Bunkyo-ku, Tokyo 113-8656, Japan; 3 Advanced Institute for Materials Research, Tohoku University, 2-1-1 Katahira, Aoba-ku, Sendai 980-8577, Japan; 4 Laboratory for Nanoelectronics and Spintronics, Research Institute of Electrical Communication, Tohoku University, 2-1-1 Katahira, Aoba-ku, Sendai 980-8577, Japan; 5 Center for Spintronics Integrated Systems, Tohoku University, 2-1-1 Katahira, Aoba-ku, Sendai 980-8577, Japan; 6 Center for Innovative Integrated Electronic Systems, Tohoku University, 468-1 Aramaki Aza Aoba, Aoba-ku, Sendai 980-0845, Japan; 7 Center for Spintronics Research Network, Tohoku University, 2-1-1 Katahira, Aoba-ku, Sendai 980-8577, Japan

## Abstract

Polycrystalline metal oxides find diverse applications in areas such as nanoelectronics, photovoltaics and catalysis. Although grain boundary defects are ubiquitous their structure and electronic properties are very poorly understood since it is extremely challenging to probe the structure of buried interfaces directly. In this paper we combine novel plan-view high-resolution transmission electron microscopy and first principles calculations to provide atomic level understanding of the structure and properties of grain boundaries in the barrier layer of a magnetic tunnel junction. We show that the highly [001] textured MgO films contain numerous tilt grain boundaries. First principles calculations reveal how these grain boundaries are associated with locally reduced band gaps (by up to 3 eV). Using a simple model we show how shunting a proportion of the tunnelling current through grain boundaries imposes limits on the maximum magnetoresistance that can be achieved in devices.

Electrically insulating thin metal oxide films are a key functional element in diverse technologies including in spintronics^1^, microelectronics^2^, photovoltaics^3^, optoelectronics^4^, sensing^5^ and catalysis^6^. While in almost all practical devices metal oxide films are polycrystalline, very little is known about the effect of grain boundaries on their electrical properties. This contrasts with the situation for point defects in thin films, such as vacancies and impurities, whose role in trap assisted tunnelling processes has been well studied both experimentally and theoretically^7^,^8^,^9^,^10^. One of the reasons grain boundaries (GBs) in oxide films are so poorly understood is that it is extremely challenging to probe the structure of such buried interfaces directly. While atomic resolution is routinely achieved for metal supported thin oxide films using scanning tunnelling microscopy (STM), most studies have focused on relatively idealised films and the structure of GBs has not been resolved^11^,^12^,^13^. On the other hand, the structure of GBs in bicrystals has been the subject of extensive investigation by atomic resolution scanning transmission electron microscopy (STEM)^14^,^15^,^16^,^17^,^18^. In many cases atomic resolution of the GB defects in bicrystals is achieved but similar observations for thin film samples have proved challenging to obtain^19^,^20^,^21^. The absence of detailed information on the atomic structure of GBs in oxide films also presents an obstacle to theoretical modelling of associated electronic properties. While in principle one could attempt to predict structures from first principles this is made challenging by the fact the films are often grown and post-processed in highly non-equilibrium conditions meaning predictions based on structures with low formation energies may have little to do with reality^22^,^23^,^24^,^25^. This absence of direct evidence continues to fuel speculation on the role of GBs as preferential electron tunnelling pathways and sinks for the segregation of point defects^26^,^27^.

In this study, we employ annular bright-field (ABF) and annular dark-field (ADF) imaging with an advanced STEM microscope (JEOL JEM-ARM200F) installed with a spherical aberration corrector (CEOS GmbH) to resolve the atomic structure of frequently occurring GBs within an ultrathin MgO film buried inside a multilayer stack. In the following we present ABF images only as we find little difference between the resolved structures in this material. The particular device we consider is a magnetic tunnel junction (MTJ) which finds applications as a magnetic sensor in hard disk read heads and in emerging non-volatile magnetic random access memory (MRAM) technologies^28^,^29^,^30^,^31^,^32^. The active part of the device consists of an insulating MgO film sandwiched between two ferromagnetic FeCoB layers^25^,^33^,^34^. The MgO film is polycrystalline, with a typical grain size of 10–15 nm with each grain oriented such that the [001] crystallographic direction is parallel to the film normal. The presence of GBs in these films has long been known from cross-sectional TEM imaging^20^. However, atomic level imaging of the structure of GBs in films has not been obtained since the [001] texture of the films means that atomic columns at GBs are unlikely to be aligned with the imaging direction. To image these GBs with atomic resolution we prepare samples for plan-view imaging by stripping all layers from the multilayer stack leaving only the MgO layer (e.g. see Fig. 1a). Analysis of the STEM images we obtain reveals two commonly occurring structural unit types which correspond to an asymmetric tilt GB (which would not have been predicted in the absence of direct imaging due to its relatively high formation energy) and a more common symmetrical tilt GB^35^. On the basis of these results we perform first principles theoretical modelling of the structure and electronic properties of these GBs. We find both GBs introduce a number of interface states inside the MgO gap and we elucidate their spatial localisation around structural units at the GBs. A consequence of these defect states is that we predict the local barrier to electron tunnelling is effectively reduced by up to 3 eV at GBs, an important consequence for the physics of MTJ devices. In particular, the shunting of a proportion of the tunnelling current through GBs rather than though the bulk of MgO grains is predicted to reduce the magnitude of the tunnelling magneto-resistance (TMR)^29^,^36^,^37^,^38^. Removing GBs or passivating the associated interface states should therefore improve the performance of MTJs. More generally, these results highlight how by combining advanced imaging and first principles theoretical calculations one can gain invaluable insight into the effect of GBs on the electronic properties of thin oxide films and guide improvements in device performance.

## Results

**Figure 1:**
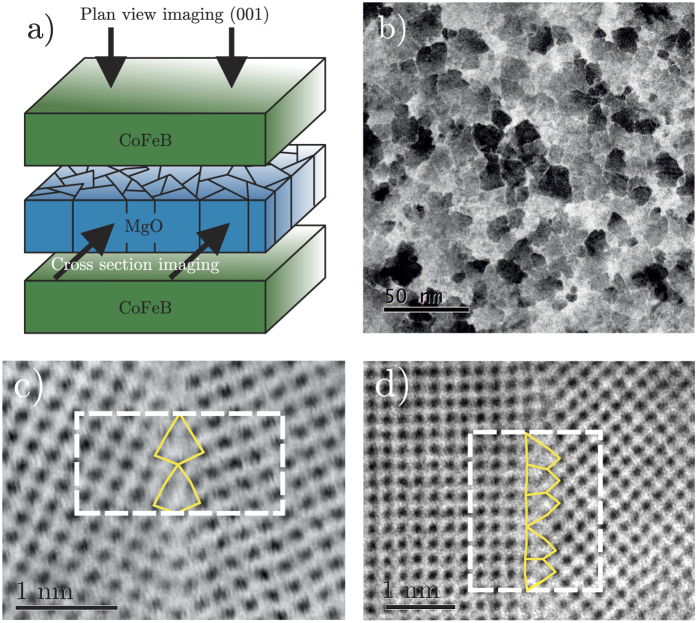
ABF-STEM images of MgO polycrystalline samples. (**a**) Schematic showing the magnetic tunnel junction investigated in this study with cross-sectional and plan-view imaging directions indicated. (**b**) ABF-STEM images showing the nanometre scale granular structure of the MgO films. (**c,d**) Examples of commonly occurring structural units at grain boundaries corresponding to segments of a Σ5(210)[001] symmetric tilt grain boundary (**c**) and a (100)/(110)[001] asymmetric tilt grain boundary (**d**). The white dashed boxes indicate the periodic supercells which are used to model these grain boundary defects.

A schematic of the MTJ device that is the focus of this investigation is shown in Fig. 1a. We prepare a sample for plan view imaging with STEM that consists of only the MgO layer from this device. Usually a TEM sample should be less than around 10 nm in thickness in order to be transparent to the electron beam. Preparation of the 10 nm thick plan-view sample which consists of only the MgO thin film is achieved using a back-thinning method involving mechanical dimpling and Ar-milling (see Methods). As shown in Fig. 1b the film is polycrystalline with irregularly shaped grains with typical diameters in the range 10 to 15 nm. Atomic columns in the [001] crystallographic direction are observed in every grain indicating that the film is almost perfectly [001] textured (see Supplementary Information Fig. 2). The interfaces between grains (i.e. grain boundaries) consist of a connected chain of GB structural units (SUs). We have analysed 35 images like that shown in Fig. 1b to identify the most frequently occurring GB SUs in the MgO film (see Supplementary Information Figs 3–5). The most frequently occurring SU has a triangular shape with mirror symmetry along the plane of the GB (shown in Fig. 1c). Chains of SUs of this type are analogous to segments of Σ5(210)[001] symmetric tilt GBs. Figure 1d shows the second most commonly occurring SU which consists of a two back-to-back (or top to top) triangular shapes without mirror symmetry about the GB plane. Chains of SUs of this type are analogous to segments of (100)/(110)[001] asymmetric tilt GBs (i.e. the grain on the left is rotated 45° with respect to the grain on the right). The observation of GBs of this type is quite unexpected due to the high energy associated with asymmetric tilt GBs.

**Figure 2:**
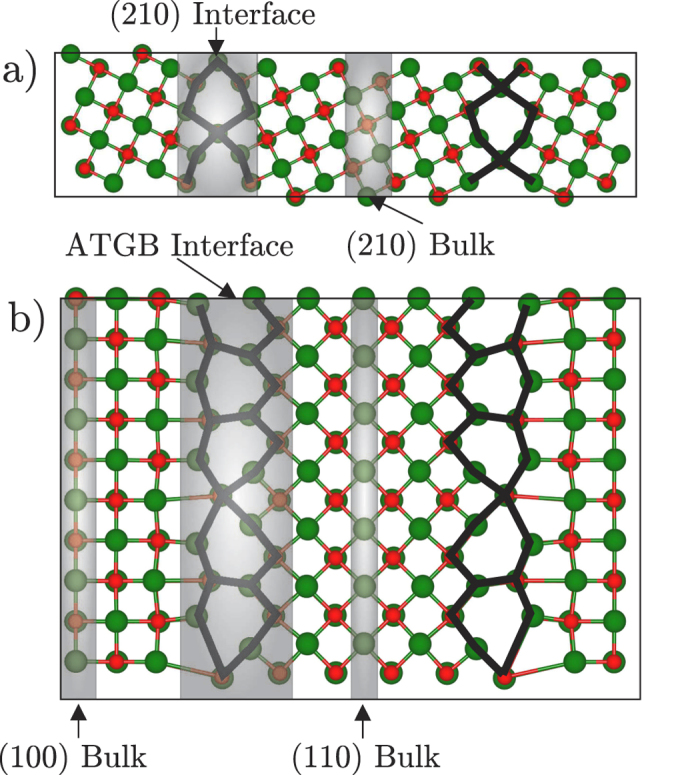
Theoretical models of MgO thin films. Supercells used to model commonly occurring grain boundary structures present in MgO films. (**a**) Σ5(210)[001] symmetric tilt grain boundary and (**b**) (100)/(110)[001] asymmetric tilt grain boundary. Red and green atoms represent O and Mg respectively. Shaded areas signify which atoms are used to produce the projected density of states shown in Fig. 4.

**Figure 3:**
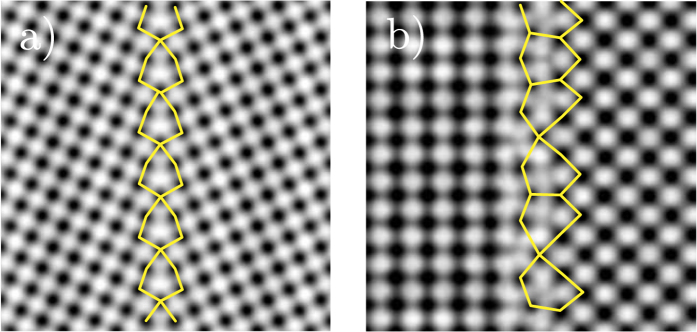
TEM image simulations of theoretical MgO models. TEM image simulations based on first principles theoretical models. a) Σ5(210)[001] symmetric tilt grain boundary and b) (100)/(110)[001] asymmetric tilt grain boundary.

To provide insight into the structure and stability of these GBs we perform first principles calculations using density functional theory (DFT) (see Methods). We construct three-dimensionally periodic supercells to model the Σ5(210)[001] symmetric tilt GB and the (100)/(110)[001] asymmetric tilt GB. These models do not seek to replicate the complex structures shown in Fig. 1b but to describe the individual SUs in Fig. 1c,d. In the former case it is straightforward to construct such a supercell using methods previously described for modelling symmetric tilt GBs in a range of oxide materials including MgO, HfO_2_, TiO_2_ and SrTiO_3_^24^,^26^,^39^. In the latter case the noncommensurate nature of the (100) and (110) orientations makes constructing a supercell more challenging. Here, we model this GB by constructing an interface between 7 unit cells of MgO(100) and 5 unit cells of MgO(110) resulting in only ±0.5% strain in each grain (see Methods). Figure 2 shows the optimised atomic structure of the two GB models. For the Σ5(210)[001] GB our computational search identifies two stable structures with similar energy which differ only by a rigid translation of one grain with respect to the other. Here we focus our attention on the model which matches the experimental images. This is in fact a slightly less favourable structure according to DFT (by 0.4 J/m^2^) however the non-equilibrium nature of the growth or impurity segregation (as discussed in previous work on MgO^22^) could influence the GB structure which is formed in reality. The formation energy of the Σ5(210)[001] GB is calculated to be 1.7 J/m^2^. The formation energy of the (100)/(110)[001] asymmetric tilt GB is much higher as expected 2.2 J/m^2^. We have performed electron microscopy image simulations (Fig. 3) for these two supercells and the agreement with the experimental ABF-STEM images is very good, giving us confidence that these structures are realistic representations of the GB structures present in the MgO films.

**Figure 4:**
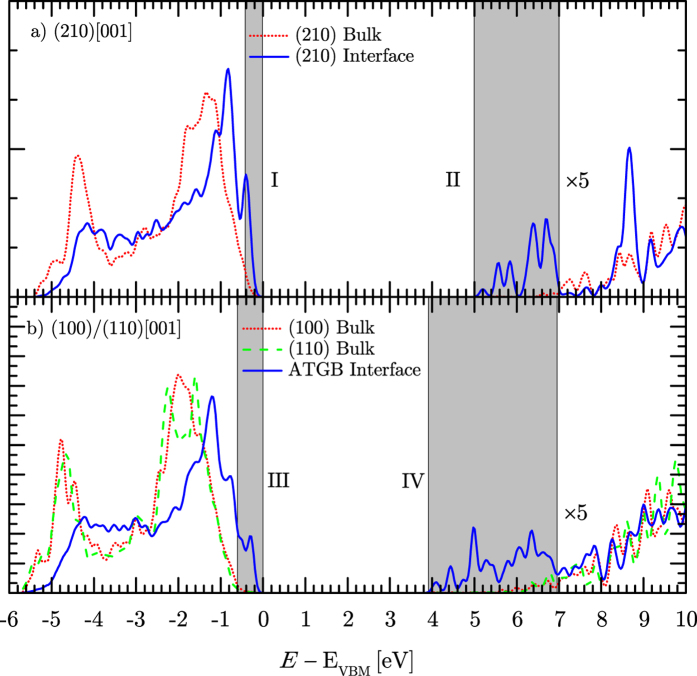
Density of states near MgO interfaces. Density of states of the (**a**) Σ5(210)[001] symmetric tilt grain boundary and (**b**) (100)/(110)[001] asymmetric tilt grain boundary calculated using the HSE06 functional. The DOS is projected onto bulk and interface regions (defined in Fig. 2). *E*_VBM_ is the energy of the bulk valence band maximum. Roman numerals indicate regions of interfacial electronic states. The “×5” indicates the factor the DOS of the unoccupied states have been increased by to aid visualisation.

With atomic models of the GB structures determined we now turn to predict the associated electronic properties. For each GB supercell we compute the electronic density of states (DOS) using both the semi-local exchange correlation functional (PBE) and an exchange correlation functional including non-local exact exchange (HSE06). However, the latter gives a much more accurate prediction of the band gap of bulk MgO 7.2 eV (4 eV PBE) compared to the experimental value of 7.8 eV^40^ and so is used for the results presented here. We project the DOS onto bulk and interface regions of the supercell (see shaded areas in Fig. 2) in order to isolate the electronic states associated with the GB defects. Figure 4a shows the DOS for the Σ5(210)[001] symmetric tilt GB. We find that the highest occupied electronic states in the supercell are associated with the GB (about 0.05 eV higher than the bulk valance band maximum). However, the most prominent feature is a wide band of states in a 2 eV (1 eV PBE, see Supplementary Information Fig. 7) window below the bulk conduction band minimum. This effectively reduces the band gap at the GB to 5 eV (3 eV PBE, see Supplementary Information Fig. 7), a reduction of 30% (25% PBE). For the (100)/(110)[001] asymmetric tilt GB the effects are even more striking. In this case we project onto the bulk region of each grain separately since due to the small unphysical strain in the supercell the DOS in the two bulk regions may in principle be different. However, as can be seen the difference is very small confirming that this strain has a negligible effect on predicted electronic properties. In the (100)/(110)[001] GB we also find that the highest occupied electronic states in the supercell are associated with the GB (about 0.2 eV higher than the bulk valance band maximum). However, again the most prominent feature is a wide band of states this time spanning a 3 eV (2 eV PBE, see Supplementary Information Fig. 7) window below the bulk conduction band minimum. This reduces the band gap at the GB to 4 eV (2 eV PBE, see Supplementary Information Fig. 7), a reduction of 45% (50% PBE).

**Figure 5:**
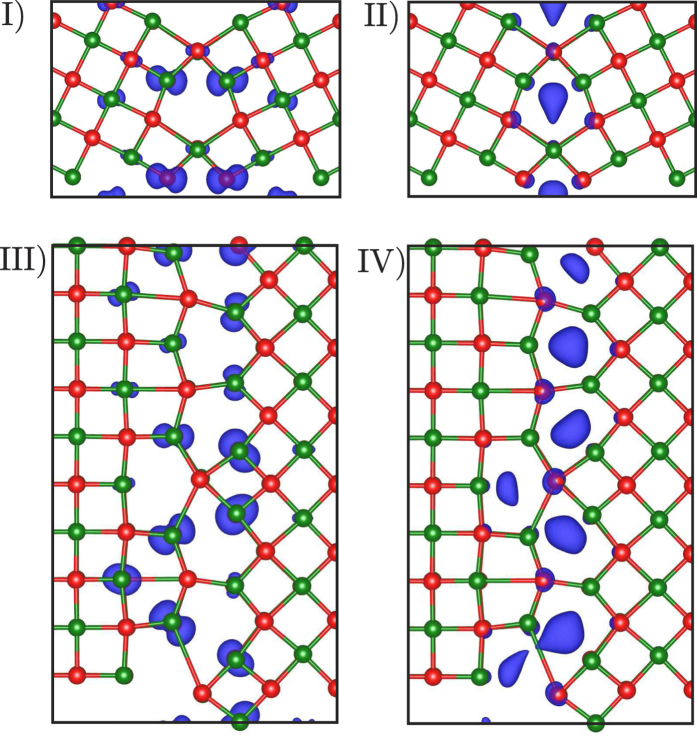
Localisation of the charge density near MgO interfaces. Total charge density (norm of the eigenfunctions) associated with electronic states in particular energy windows for the Σ5(210)[001] symmetric tilt grain boundary and (100)/(110)[001] asymmetric tilt grain boundary in MgO. The energy windows (**I− IV**) are highlighted in Fig. 4. The red and green atoms represent O and Mg respectively and the charge density is represented by the blue isosurfaces.

To provide further insight into the nature of the electronic states associated with GBs we compute the total charge density (norm of the eigenfunctions) associated with electronic states in particular energy windows. Four regions of interest within the gap of bulk MgO have been identified by shaded areas and Roman numerals in Fig. 4. Figure 5 shows the charge density isosurfaces associated with electronic states in these regions. Regions I and III are associated with occupied states that are above the bulk valence band maximum in the Σ5(210)[001] and (100)/(110)[001] GBs respectively. For region I the charge density is associated primarily with five-coordinated oxygen ions near the GB while for region III it is associated with almost all interfacial oxygen ions. The states in regions II and IV fall below the bulk conduction band minimum. In both GBs they are localised inside the triangular structural units at the GB but are extended in the [001] direction (i.e. along the film normal). For the (100)/(110)[001] GB it is notable that the deepest interfacial electronic states are localised preferentially in the largest structural units.

The above results show that electronic states associated with GBs in the MgO layer of MTJs locally reduce the band gap by up to 3 eV. This reduced gap will increase the amount of tunnelling current that is shunted through GBs rather than through the bulk-like regions. Bulk MgO is associated with a very large magnetoresistance due to a symmetry filtering effect with values as high as 3400% predicted theoretically^29^,^41^. However, due to reduced symmetry the local magnetoresistance at GBs is expected to be much lower, closer to that predicted by the Julliére model (e.g. about 67% for a electrode spin polarisation of 0.5^42^). As a result of a proportion of the spin current flowing through GBs one may expect the effective TMR of a granular MgO barrier to be reduced relative to a single crystal barrier. In the following we provide a simple model to describe this effect.

We first consider the situation in which the magnetisation in both ferromagnetic electrodes is aligned parallel and relate the resistance of the bulk and GB regions to their respective cross-sectional areas (*A*_b/gb_) and transmission coefficients (*T*_b/gb_) using the Landauer formula^43^,


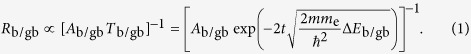


**Figure 6:**
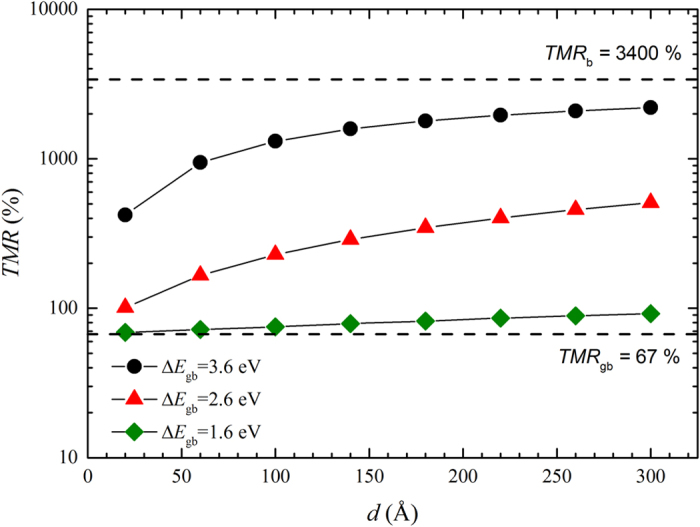
Dependence of TMR with grain thickness. Predicted dependence of the total magnetoresistance of granular FeCoB/MgO/FeCoB magnetic tunnel junctions on average MgO grain size (*d*) and the energy difference between the electrode Fermi energy and lowest unoccupied bands at the grain boundary (Δ*E*_gb_). The bulk and grain boundary TMR values employed in the model are also indicated by dashed lines.

On the right hand side of this equation we have used a simple square barrier tunnelling model for the transmission coefficient in which Δ*E*_b/gb_ is the difference between the electrode Fermi energy and the lowest unoccupied bands in the respective region, *m* is the effective mass, *m*_e_ is the mass of the electron and *t* is the thickness of the MgO layer (see Supplementary Information). Using Eq. 1 it is straightforward to calculate the effective resistance of the film, and by taking values for the TMR of the bulk and GB regions (*TMR*_b/gb_) as parameters the effective magnetoresistance of the granular film can be determined numerically. To calculate the cross-sectional areas we assume cylindrical grains of average diameter *d* separated by GBs of width 2*δ* (here we consider *δ* = 2 Å). For the other parameters we consider *m* = 1, Δ*E*_b_ = 3.6 eV (corresponding to mid-gap band alignment), *t* = 8.424 Å (corresponding to 4 monolayers of MgO), *TMR*_b_ = 3400% and *TMR*_gb_ = 67%. Figure 6 shows how the effective magnetoresistance varies with both average grain size and Δ*E*_gb_. If Δ*E*_gb_ is the same as in the bulk (3.6 eV), corresponding to an absence of GB gap states, we predict very high TMR values are possible, in excess of 1600% for typical grain sizes of around 15 nm. However, a reduction of Δ*E*_gb_ by 1 or 2 eV (expected based on the first principles calculations above) leads to a drastic decrease in the predicted magnetoresistance to around 300% and 80% respectively. For Δ*E*_gb_ = 2.6 eV the model predicts one would need to increase the average grain size to 270 nm to realise TMR values in excess of 2000%. It should be noted that the simple expression employed in Eq. 1 for the transmission coefficient does not take into account details of the MgO and Fe band structures^29^,^41^. However, since Eq. 1 is used only to determine the relative currents flowing in the bulk and GB regions and not to calculate the TMR directly we believe it offers useful qualitative or semi-quantitative insight into role GBs play in limiting the tunnelling magnetoresistance achievable in MTJ devices.

## Discussion

We have characterised the structural and electronic properties of two representative GBs which exhibit structural units which are by far the most common as determined by a detailed analysis of ABF-STEM images. Given the [001] texture of the films the presence of the symmetric Σ5(210)[001] tilt GB is consistent with its relatively low formation energy and high site coincidence. The asymmetric (100)/(110)[001] tilt GB on the other hand was unexpected and is comparatively much less stable. This can be rationalised since the observed MgO microstructure is likely a result of highly non-equilibrium growth and post processing processes. The MgO films studied here are grown on amorphous FeCoB substrates which are then annealed, removing the boron and crystallising the FeCoB electrodes^25^. Interfaces between grains in the MgO film follow complex paths (Fig. 1b) with small sections of planar GBs which are very difficult to model using first principles methods. However, since the structural units that compose these more complex GBs are similar to that in the planar GBs we believe our computational models to be representative.

Calculations for the electronic properties of the GBs have been performed using the hybrid HSE06 functional. This approach predicts the band gap of bulk MgO within 0.6 eV of the experimental value, much better than standard local or semi-local functionals (typically underestimated by about 3 eV). Therefore, it should be a reliable approach for characterising the nature of the interface states. For both types of GB we find a relatively shallow splitting of interface states near the valence band maximum but a much deeper splitting of interface states below the conduction band minimum. This asymmetry is consistent with previous results for GBs in similar oxide materials such as HfO_2_^26^,^44^. The use of PBE or HSE06 XC functionals does not change the qualitative result (i.e. that grain boundaries locally reduce the band gap, see Supplementary Information Fig. 7).

Here, we have focused on the electronic properties of pristine grain GBs however in reality it is likely that they may act as sites for the preferential segregation of intrinsic defects and impurities^22^. In general such defect segregation would increase the number of electronic states in the gap further deteriorating the insulating properties. EELS analysis on similar MTJ samples from previous work indicates that there is no significant concentration of impurities such as Fe, Co and B but we cannot completely exclude the possibility^25^,^45^. The role of defect and impurity segregation and diffusion is an issue of interest for further study but is beyond the scope of the present work.

Finally we have presented a phenomenological model to estimate the effect of enhanced GB conduction on the magnetoresistance of MTJs. The simplistic nature of the approximations means that quantitative predictions are not possible but semi-quantitative trends should be reliable. In particular we predict that due to the comparatively high conductivity of GBs a high proportion of the tunnelling current is shunted through GBs even for relatively large grain sizes. Due to the lower symmetry the current passing through GBs does not contribute a very high magnetoresistance effect leading to a substantially lower effective magnetoresistance overall. More advanced electron transport calculations, for example using the recursive Green’s function approach, would allow the effect of GBs on transport properties to be more precisely determined, but would be extremely computationally demanding owing to the large size of the supercells required^36^,^46^.

## Conclusions

To summarise we have used ABF-STEM imaging in order to resolve the granular structure of the MgO layer in FeCoB/MgO/FeCoB MTJs. For the first time we have demonstrated the possibility to prepare plan-view samples allowing us determine the atomic scale structure of GBs in highly textured films. Based on an analysis of these images we have identified commonly occurring GB structural units which we have used to construct models for first principles calculations. In this way we have been able to characterise the electronic properties of buried GB defects inside a MTJ showing that they locally reduce the MgO band gap by up to 3 eV. Finally using a simple model we show the consequences of a reduced band gap and increased conductivity for the performance of the MTJ device. More generally this study shows that combining first principles modelling and state of the art electron microscopy can provide real insight into the structural properties of thin films. Such an approach could be expanded to films of other types to understand how the atomic structure changes with different material compositions.

## Methods

### Film Fabrication

The film is deposited onto a thermally oxidised Si substrate by rf-magnetron sputtering at room temperature. The stack structure is, from the substrate side Ta(5)/ Ru(10)/Ta(5)/Fe_60_Co_20_B_20_(5)/MgO(20)/Fe_60_Co_20_B_20_(1), where numbers in parentheses are nominal thickness in nm. The bottom Ta/Ru/Ta/FeCoB serves as a seed to form the [001] textured structure of MgO, which is removed during the back-thinning process (see below). The top FeCoB deposited to protect the MgO layer is thin enough not to affect the observation of MgO structure. After the deposition, the film is annealed at 500 °C for one hour. The film fabrication method produces highly crystalline samples (with TMR as high as 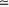
300%) as demonstrated in earlier papers by the authors^25^,^47^ (See Supplementary Information Fig. 6).

### Electron microscopy

In order to observe only the GBs of the MgO in the TMR device, we have to prepare a plan-view TEM sample which consists of only MgO. Preparation of the plan-view sample can be achieved using a back-thinning method. First, the substrate of the specimen is ground with sand paper and dimpled mechanically from only the back side (substrate side) to reduce the thickness to less than around 20 *μ*m. Then the specimen is thinned to around 10 nm using Ar-ion milling with an acceleration voltage of 1.5–3.5 kV and an incident beam angle of 4–6 deg from only the back side. Finally, the plan-view specimen which consists of only of MgO is obtained.

The GB microstructures are experimentally characterised in detail using annular bright-field (ABF) STEM. ABF-STEM imaging, whereby an annular detector is positioned within the bright field region in an atomic resolution STEM, has recently been shown to produce images showing both light and heavy element columns simultaneously^48^,^49^. The ABF-STEM images are taken with the JEOL JEM-ARM200F (Cold FEG) electron microscope with Cs-corrector (CEOS GmbH), which is operated at 200 kV. ABF-STEM imaging was performed with a probe convergence angle of 22 mrad and a detector semiangle within 11–22 mrad. The obtained STEM images were low-pass filtered to reduce a high frequency noise.

### Theoretical calculations

GB structures are modelled using bicrystal supercells constructed on the basis of structural unit geometries frequently observed in the STEM images. The two GBs we consider are the Σ5(210)[001] symmetric tilt GB and the (100)/(110)[001] asymmetric tilt GB. The former contains 152 atoms and the latter 266 atoms. For the asymmetric tilt GB it is necessary to impose a small strain of +0.5% on MgO(100) and −0.5% on MgO(110) in order to accommodate the two grains in a periodic supercell. For both GBs stable structures are determined by optimising the total energy with respect to the position of all atoms and translation of one grain relative to the other. We first perform optimisations using an interatomic potential before refining structure at the density functional theory (DFT) level as explained in detail in previous work^44^,^50^.

The first principles DFT calculations have been performed using the Vienna ab-initial simulation package (VASP)^51^. We applied the projector augmented-wave method^52^ and a plane wave cut-off energy of 350 eV. Geometry optimisation was performed using the GGA functional and a 1 × 6 × 3 Gamma point centred k-point grid for the Σ5(210)[001] GB and a 1 × 6 × 1 grid for the (100)/(110)[001] GB. All atoms in the supercells were fully optimised with respect to interatomic force tolerance of 0.01 eV per Å. Single point calculations were performed on optimised structures using the hybrid HSE06 functional to determine the electronic density of states and charge density isosurfaces. The supercells were rescaled according to the bulk MgO optimised lattice constants obtained using the HSE06 functional to minimise strain, then electronically converged.

## Additional Information

**How to cite this article:** Bean, J. J. *et al*. Atomic structure and electronic properties of MgO grain boundaries in tunnelling magnetoresistive devices. *Sci. Rep.* **7**, 45594; doi: 10.1038/srep45594 (2017).

**Publisher's note:** Springer Nature remains neutral with regard to jurisdictional claims in published maps and institutional affiliations.

## Supplementary Material

Supplementary Information
