# Supplementary material for: Atomic structure and electronic properties of MgO grain boundaries in tunnelling magnetoresistive devices
Source: Sci Rep. 2017 Apr 4;7:45594. doi: 10.1038/srep45594 (PMC5379487; doi:10.1038/srep45594)
Supplement: Supplementary Information [file srep45594-s1.pdf]

Supplemental Information: Atomic structure and electronic properties of MgO grain boundaries in tunnelling magnetoresistive devices

Jonathan J. Bean,[1] Mitsuhiro Saito,[2,3] Shunsuke Fukami,[4,5,6,7] Hideo Sato,[5,6,7] Shoji Ikeda,[5,6,7] Hideo Ohno,[3,4,5,6,7] Yuichi Ikuhara,[2,3] and Keith P. McKenna[8]

[1]*Department of Physics, Heslington, York, North Yorkshire, YO10 5DD, U.K.*

[2]*Institute of Engineering Innovation, The University of Tokyo, 2-11-16 Yayoi, Bunkyo-ku, Tokyo 113-8656, Japan*

[3]*Advanced Institute for Materials Research, Tohoku University, 2-1-1 Katahira, Aoba-ku, Sendai 980-8577, Japan*

[4]*Laboratory for Nanoelectronics and Spintronics, Research Institute of Electrical Communication, Tohoku University, 2-1-1 Katahira, Aoba-ku, Sendai 980-8577, Japan*

[5]*Center for Spintronics Integrated Systems, Tohoku University, 2-1-1 Katahira, Aoba-ku, Sendai 980-8577, Japan*

[6]*Center for Innovative Integrated Electronic Systems, Tohoku University, 468-1 Aramaki Aza Aoba, Aoba-ku, Sendai 980-0845, Japan*

[7]*Center for Spintronics Research Network, Tohoku University, 2-1-1 Katahira, Aoba-ku, Sendai 980-8577, Japan*

[8]*Department of Physics, Heslington, York, North Yorkshire, YO10 5DD*

Analysis of grain boundary structures

We have analysed the atomic structure of grain boundaries (GBs) appearing in the MgO thin films from 35 scanning transmission electron microscopy (STEM) images of different regions of the sample. We have identified a number of commonly occurring structural units (SUs) which are shown in Fig. . We find by far the most frequently occurring SU has a triangular shape with mirror symmetry along the plane of the GB. Chains of SUs of this type are analogous to segments of $\Sigma 5(210)[001]$ symmetric tilt GBs. There is also a considerable number of SUs which involve two back-to-back (or top-to-top) triangular shapes without mirror symmetry about the GB plane. Chains of SUs of this type are analogous to segments of (100)/(110)[001] asymmetric tilt GBs (i.e. the grain on the left is rotated 45° with respect to the grain on the right). Some of the STEM images used in this analysis are shown in Figs. 3-5, with identified SUs indicated.

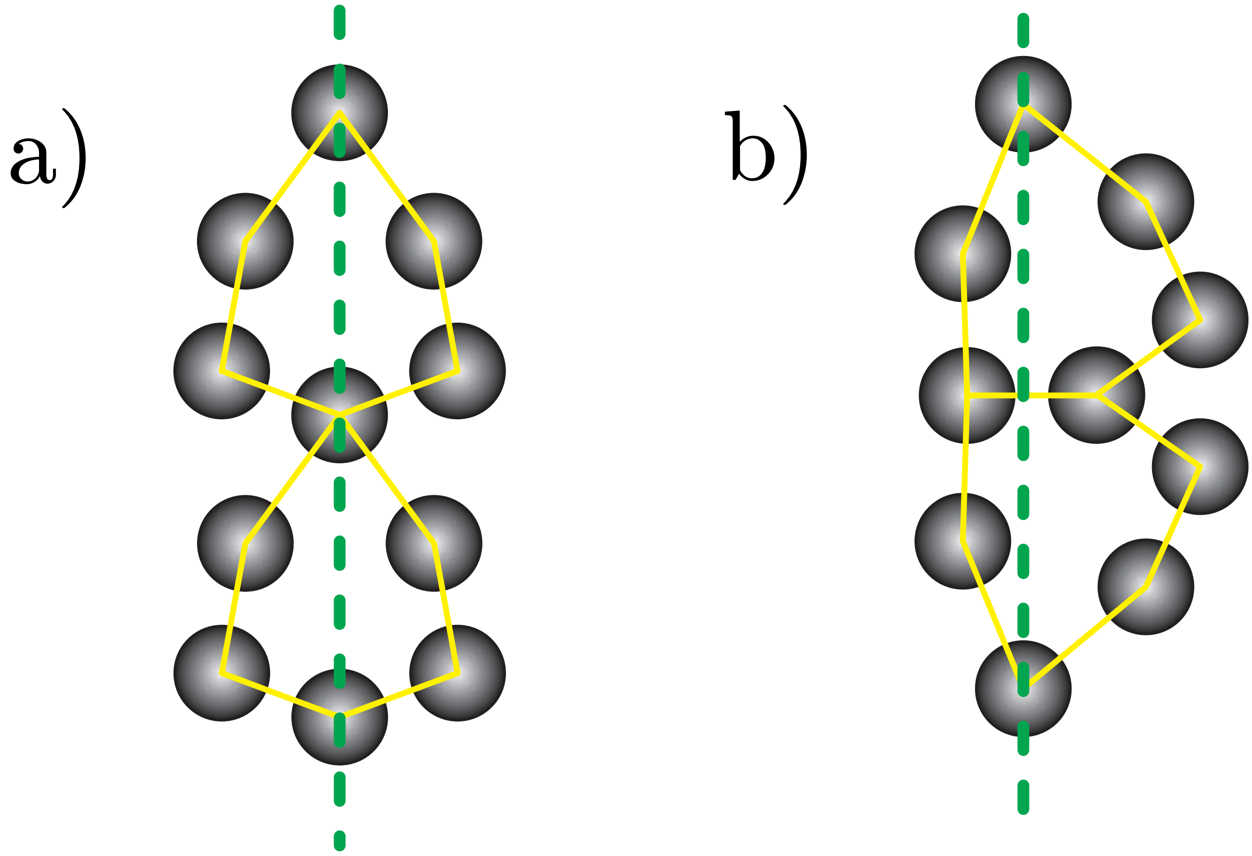

FIG. 1: Two commonly occurring structural units in the MgO films. a) a-SU which has a triangular shape with mirror symmetry along the plane of the grain boundary. b) b-SU which involves two back-to-back (or top-to-top) triangular shapes without mirror symmetry about the grain boundary plane.

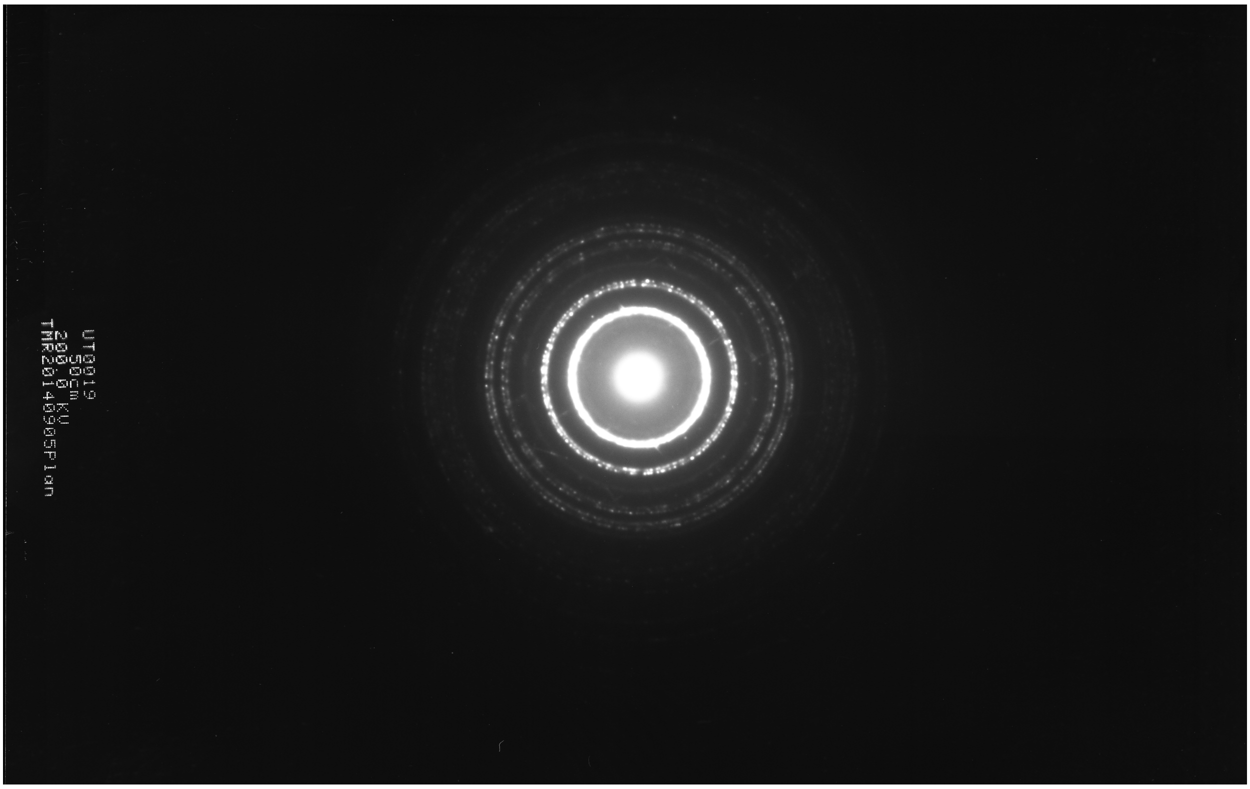

FIG. 2: Diffraction pattern from polycrystalline films along the plan-view direction. Typical ring patterns indicate a polycrystalline sample with each ring split by several percent due to lattice mismatch between MgO and metal.

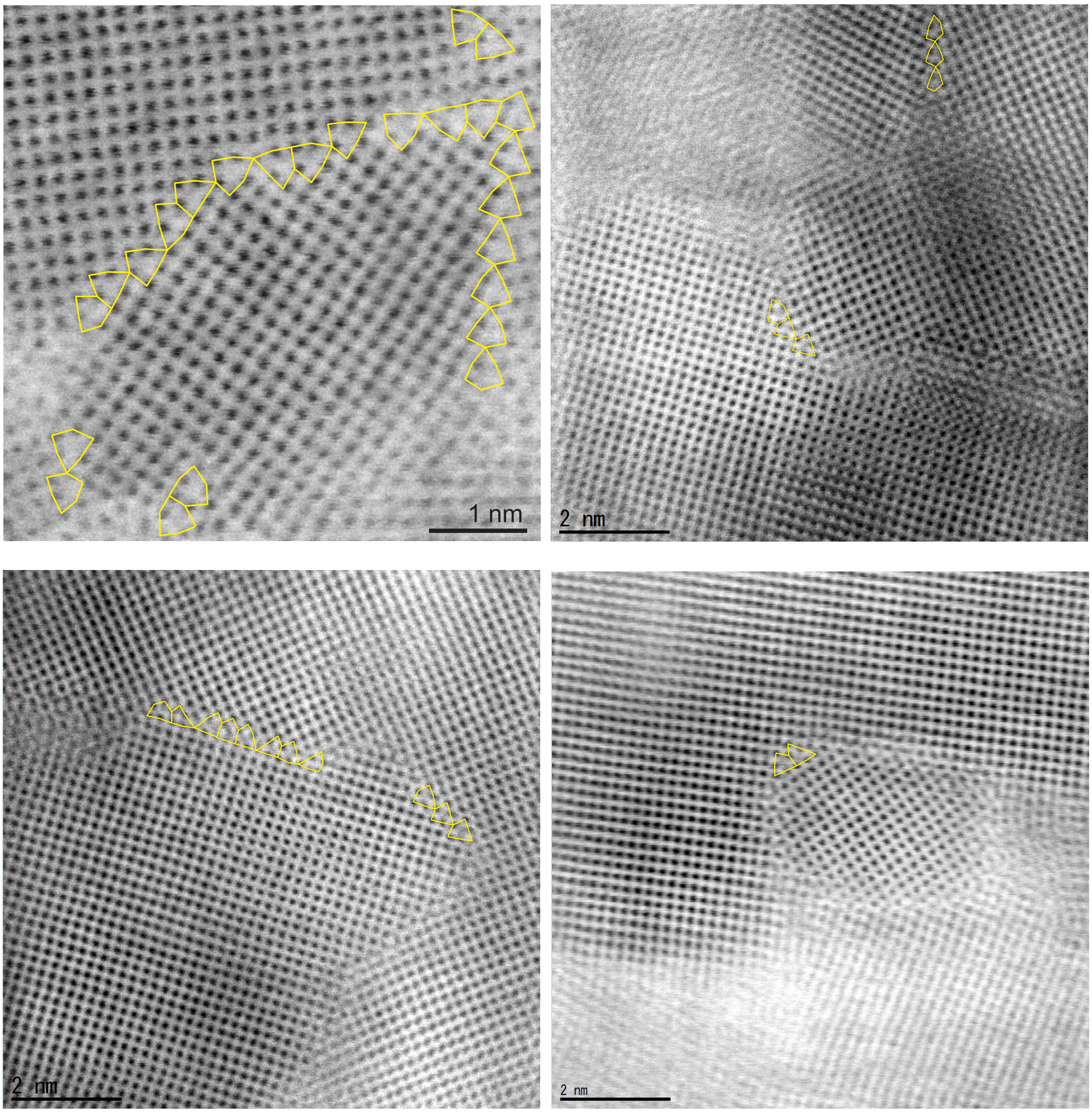

FIG. 3: Scanning transmission electron microscopy images of the MgO film with identified structural units highlighted (images 1-4).

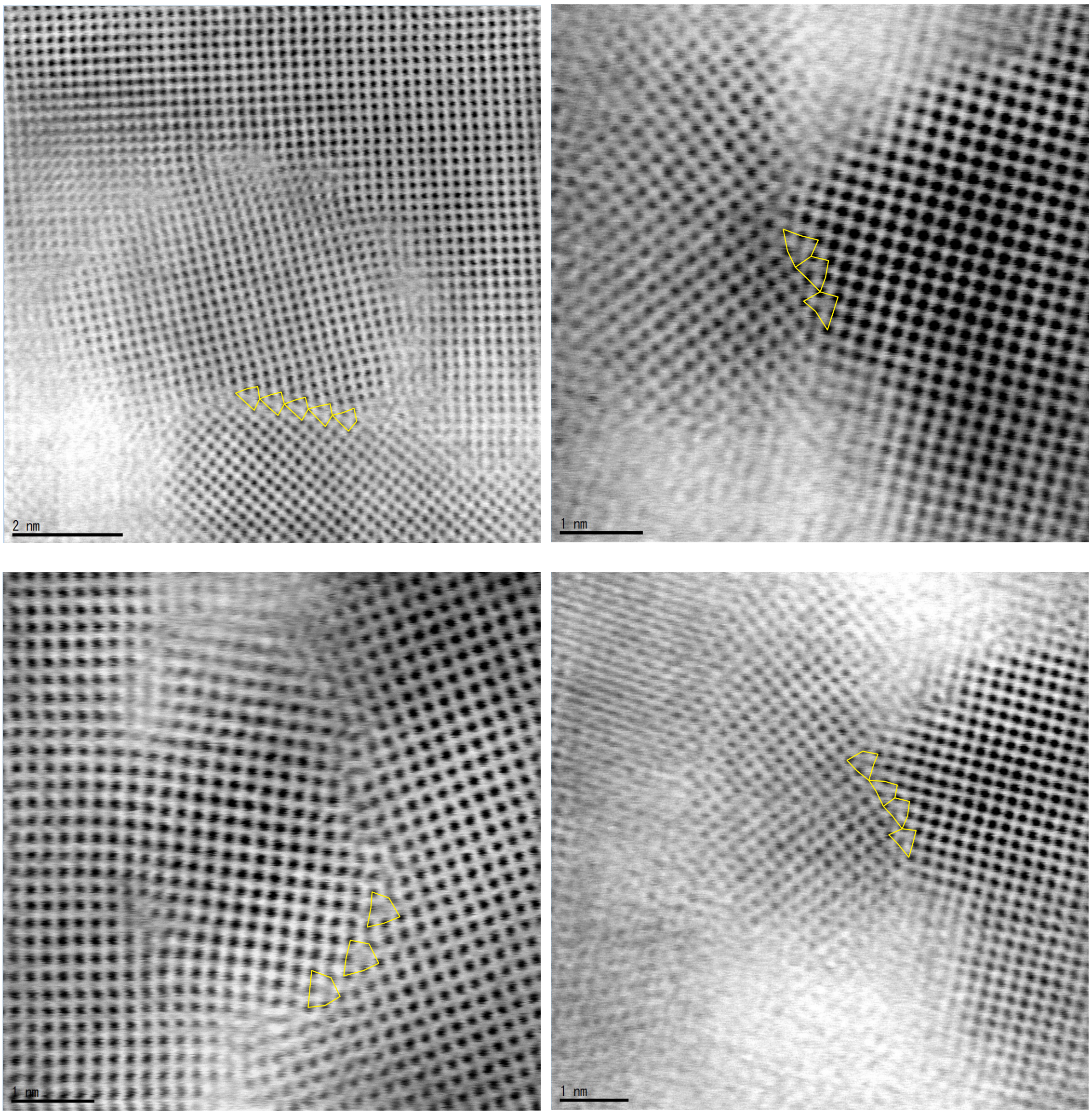

FIG. 4: Scanning transmission electron microscopy images of the MgO film with identified structural units highlighted (images 5-8).

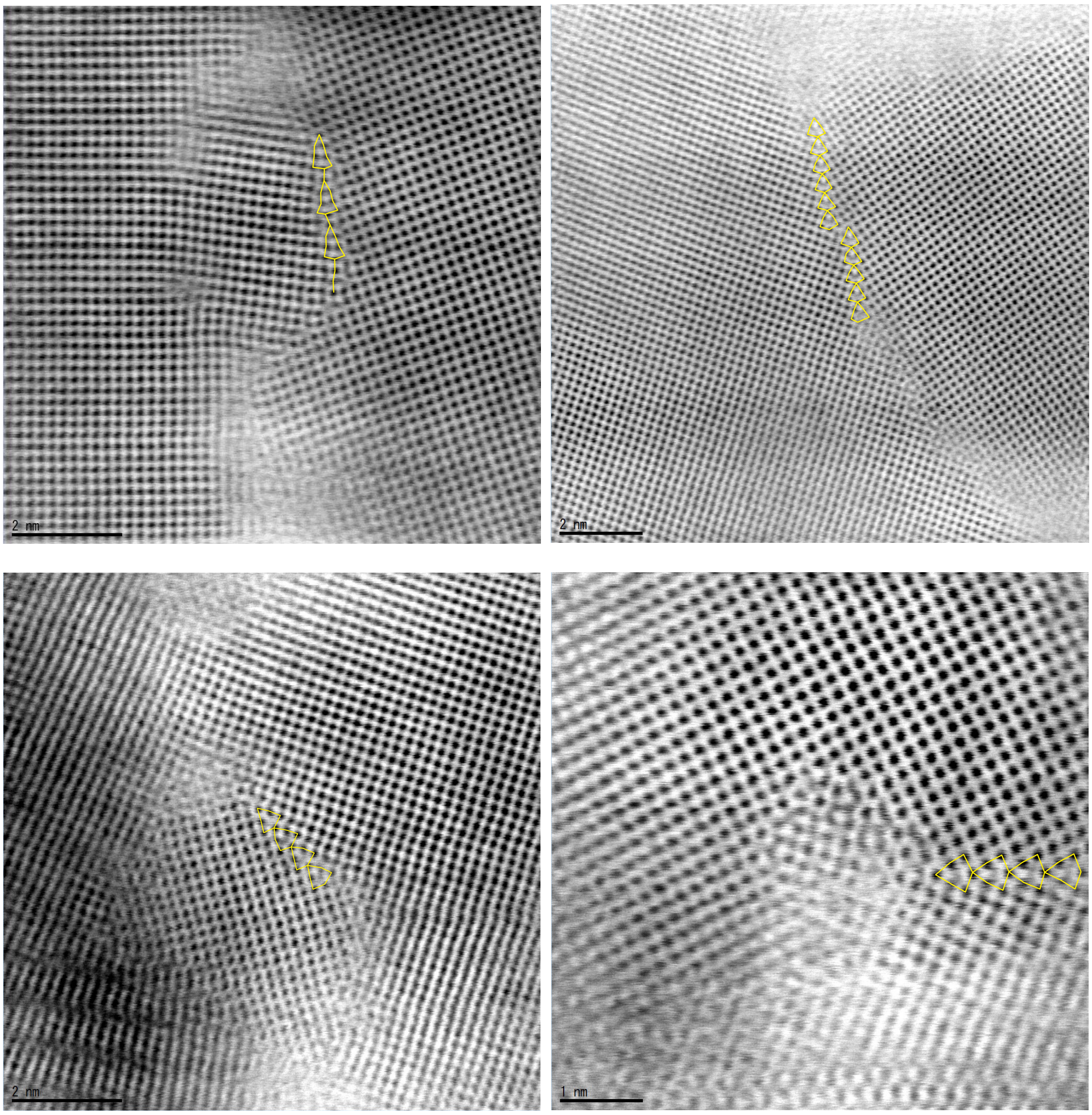

FIG. 5: Scanning transmission electron microscopy images of the MgO film with identified structural units highlighted (images 9-12).

In order to obtain a large TMR it is important to have an MgO film which is high quality and epitaxial. In Fig. 6 cross sectional images of MgO for different stack thicknesses is shown, even for large thicknesses long columnar grains are found perpendicular to the substrate. The MgO texture is largely kept during film growth indicating that there is not a strong dependence with the thickness of MgO film.

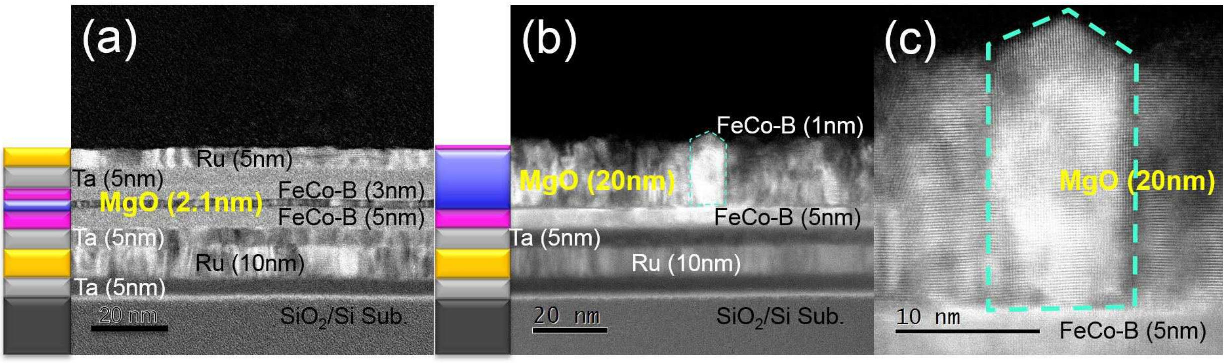

FIG. 6: Cross sectional TEM images of a magnetic tunnel junction stack for different thicknesses of MgO. a) MgO thickness of 2.1nm as used in high performance MTJs b) MgO thickness of 20nm as used in plan-view images c) Zoomed in on large epitaxial region of 20nm thick MgO.

PBE DOS

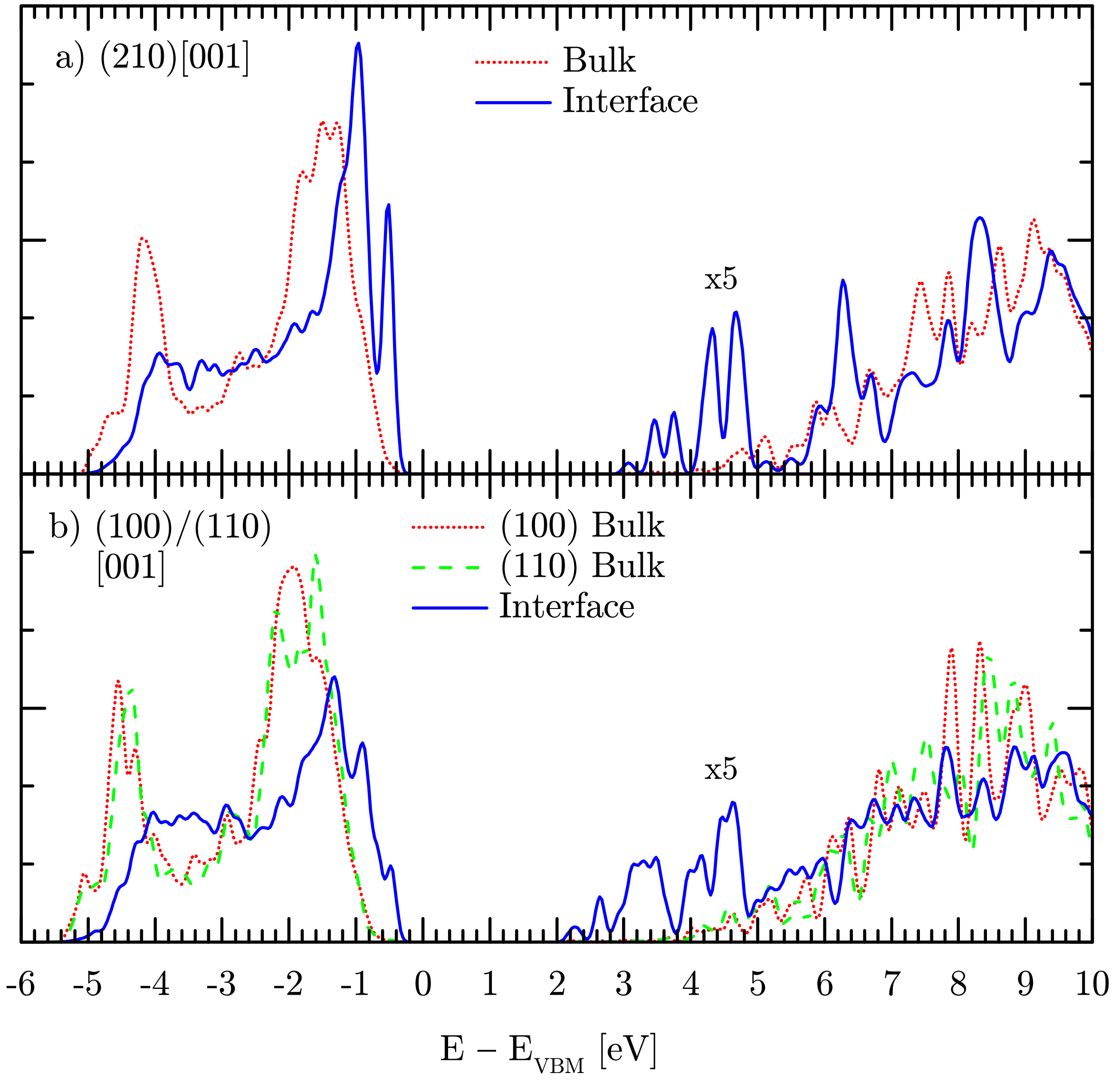

FIG. 7: Density of states of the a) Σ5(210)[001] symmetric tilt grain boundary and b) (100)/(110)[001] asymmetric tilt grain boundary calculated using the PBE functional. The DOS is projected onto bulk and interface regions. E_{VBM} is the energy of the bulk valence band maximum. The "×5" indicates the factor the DOS of the unoccupied states have been increased by to aid visualisation.

In Fig. 7 the density of states for the ATGB and STGB are shown at the PBE level. It is observed that the defect states associated with the GB closely resemble that of the HSE06

functional other than a scaling factor resulting in a smaller band gap.

Tunnelling magnetoresistance model

We first consider the situation in which the magnetisation in both ferromagnetic electrodes is aligned parallel. On application of a small electrical bias between the electrodes current will flow through the bulk-like grains and through GBs. We model this situation using a simple equivalent circuit model in which two resistors (representing the resistances of the bulk and GB conduction channels) are connected in parallel. Employing a simplification of the Landauer formula we express the resistance of each conductional channel in terms of their respective cross-sectional area ($A_{\mathrm{b/gb}}$) and average transmission coefficient ($T_{\mathrm{b/gb}}$),

$$R_{\mathrm{b/gb}} \propto \left(A_{\mathrm{b/gb}} T_{\mathrm{b/gb}}\right)^{-1}. \tag{1}$$

Taking a simple one-dimensional model for square-barrier tunnelling the transmission coefficient can be approximated as,

$$T = \exp(-2\kappa_{\mathrm{b/gb}} t), \tag{2}$$

where t is the film thickness and,

$$\kappa_{\mathrm{b/gb}} = \sqrt{\frac{2mm_{\mathrm{e}}}{\hbar^2}\Delta E_{\mathrm{b/gb}}}, \tag{3}$$

where m is the effective mass, m_{e} is the mass of the electron and $\Delta E_{\mathrm{b/gb}}$ is the difference between the electrode Fermi energy and the bottom of the lowest unoccupied band in the MgO film. Therefore, the resistances associated with the bulk and grain boundary can be written as (Eq. 1 in the main text),

$$R_{\mathrm{b/gb}} \propto \left[A_{\mathrm{b/gb}} \exp\left(-2\left(\sqrt{\frac{2mm_{\mathrm{e}}}{\hbar^2}\Delta E_{\mathrm{b/gb}}}\right) t\right)\right]^{-1}. \tag{4}$$

The total resistance of the MgO film in the case of ferromagnetic alignment of the electrode is simply given by,

$$R^{\mathrm{p}} = \frac{R_{\mathrm{b}} R_{\mathrm{gb}}}{R_{\mathrm{b}} + R_{\mathrm{gb}}}. \tag{5}$$

On reversal of the magnetization of one of the electrodes (anti-parallel alignment) the resistance of both the bulk and GB conduction channels is increased due to the tunnelling

magnetoresistance effect. The change in resistance for the bulk conduction channel is predicted to be very large owing to the special symmetry of MgO interfaced to CoFe (here we consider $TMR_{\rm b} = \Delta R_{\rm b}/R_{\rm b} = 3400$ % on the basis of previous calculations). On the other hand, the change in resistance for GBs is expected to be much lower, closer to that predicted by the Julliére model (here we consider $TMR_{\rm gb} = \Delta R_{\rm gb}/R_{\rm gb} = 67$ %). It is straightforward to numerically determine the total resistance in the case of anti-parallel alignment ($R^{\rm ap}$) by substituting into Eq. 5. Finally we define the effective magnetoresistance of the film as,

$$\mathrm{TMR_{eff}} = \frac{R^{\rm ap} - R^{\rm p}}{R^{\rm p}} = \frac{\Delta R}{R^p}. \tag{6}$$

Considering cylindrical grains separated by GBs of width 2δ (δ =2 Å and taking approximate values of other quantities from the theoretical calculations above ($m = 1$, $\Delta E_{\rm b} = 3.6$ eV and $t = 8.424$ Å) $\mathrm{TMR_{eff}}$ is calculated for different average grain diameters (d) and the results are shown in Fig. 6 (main text).
